# Second-generation antipsychotics and metabolic syndrome: a role for mitochondria

**DOI:** 10.3389/fpsyt.2023.1257460

**Published:** 2023-11-24

**Authors:** Katherine R. H. Mortimer, Mohammed Zia Ul Haq Katshu, Lisa Chakrabarti

**Affiliations:** ^1^School of Veterinary Medicine and Science, University of Nottingham, Nottingham, United Kingdom; ^2^Institute of Mental Health, School of Medicine, University of Nottingham, Nottingham, United Kingdom; ^3^Nottinghamshire Healthcare NHS Foundation Trust, Nottingham, United Kingdom

**Keywords:** antipsychotics, psychosis, mitochondria, energy metabolism, metabolic syndrome

## Abstract

Psychosis is a known risk factor for developing metabolic syndrome (MetS). The risk is even greater in patients who are taking second-generation antipsychotics (SGAs). SGAs exacerbate metabolic abnormalities and lead to a 3-fold increased risk of severe weight gain, type 2 diabetes, and cardiovascular disease in patients. Mitochondrial dysfunction is a hallmark of MetS. Mitochondria process glucose and fatty acids into ATP. If these processes are impaired, it can result in dyslipidaemia, hyperglycaemia and an imbalance between nutrient input and energy output. This leads to increased adiposity, insulin resistance and atherosclerosis. It is unclear how SGAs induce MetS and how mitochondria might be involved in this process. It has been found that SGAs impair cellular glucose uptake in liver, dysregulating glucose and fatty acid metabolism which leads to an accumulation of glucose and/or lipids and an increase reactive oxygen species (ROS) which target mitochondrial proteins. This affects complexes of the electron transport chain (ETC) to reduce mitochondrial respiration. While there is a suggestion that SGAs may interact with a variety of processes that disrupt mitochondrial function, some of the results are conflicting, and a clear picture of how SGAs interact with mitochondria in different cell types has not yet emerged. Here, we outline the current evidence showing how SGAs may trigger mitochondrial dysfunction and lead to the development of MetS.

## Introduction

### Psychosis and the use of antipsychotic medication: the discovery of first-generation antipsychotics

The term psychoses describes a cluster of psychiatric disorders which include acute and transient psychotic disorder, schizophrenia, schizoaffective disorder, delusional disorder, bipolar affective disorder and psychotic depression (1). Schizophrenia and psychosis have a lifetime prevalence of 1% and a high global economic burden (1, 2). The aetiology of psychosis is multifactorial, including genetics, substance misuse, and medical conditions, like infections, and autoimmune disorders (3–6). Psychosis is primarily treated with antipsychotic medications (APs) but a variety of comorbidities associated with psychosis means antidepressants or anti-anxiety medications are prescribed alongside (1). Prescription of antipsychotic medications doubled between 2000 and 2014 and the rate of first prescriptions of antipsychotics increased between 2000 and 2019 by 2% each year (7, 8). Antipsychotics are divided into two subclasses, first-generation antipsychotics, also known as typical antipsychotics, and second-generation or atypical antipsychotics.

First-generation antipsychotics (FGAs), were discovered due to their sedative effects and by chance were found to reduce the positive symptoms, hallucinations and delusions, of psychosis (9). FGAs such as chlorpromazine and haloperidol, were some of the first drugs to be developed and utilised in treating mental illnesses (10). Shortly after their use in clinical practise, extrapyramidal symptoms (EPS) such as tardive dyskinesia, parkinsonism and dystonia, were classed as side effects of FGA treatment (9). These symptoms were provoked by the dopamine receptor antagonism of FGAs, particularly their affinity for the D2 receptor (11). Following this, second-generation antipsychotics (SGAs) were developed, which reduce EPS as they have more diverse receptor affinities.

### Clozapine: the first second-generation antipsychotic

Clozapine was the first SGA to be developed by Wander Laboratories in 1959; it was discovered due to its similar structure to tricyclic antidepressants (12). When clozapine was found to have antipsychotic properties but no motor side effects, it became apparent that EPS were not necessary for the efficacy of antipsychotics (12). Clozapine has a more diverse receptor profile than FGAs, with an affinity for multiple serotonergic receptors, alpha-1 adrenergic receptors, D3 and D4 receptors and a lower affinity for D2 receptors (13, 14). The era of SGAs as superior alternatives to FGAs started in the 1970s, however it was cut short as within 6 months of clozapine administration to patients during clinical trials, cases of fatal agranulocytosis were reported (15). There were 17 cases in ~3,000 patients, which led to eight fatalities (16). While the risk is low, 0.8% of patients develop clozapine-induced agranulocytosis and low neutrophil levels shortly after starting clozapine treatment (17). In the mid to late 1980s, clozapine was tested against the commonly prescribed FGA chlorpromazine for tolerability and efficacy in patients (18). Clozapine showed increased efficacy in patients who were unresponsive to a previous antipsychotic, with particular focus on improving negative psychotic symptoms such as apathy and asocial behaviour, as well as positive symptoms (19, 20). Consequently, treatment with clozapine was reintroduced but now requires patients to undergo weekly blood testing and in recent years it has been reserved for patients who are deemed ‘treatment-resistant’. Treatment-resistant patients are defined by those who have not responded to two or more previously prescribed antipsychotics (1).

### The refinement of second-generation antipsychotics

Following the incidences of clozapine-induced agranulocytosis, analogues of clozapine were explored to find a drug with similar efficacy in treating psychosis, a reduced propensity for EPS but one that did not affect neutrophil levels in the blood. Olanzapine was developed by Eli Lilly and Co., with a similar receptor binding profile to clozapine but a slightly different chemical structure and presented a promising development in the field of antipsychotic medication (21). Olanzapine has shown to be efficacious in treating first-episode psychosis with a lower discontinuation rate than haloperidol and distinctly does not induce agranulocytosis like clozapine (21). Clinical trials in the late 1990s showed olanzapine treatment produced a greater reduction in positive and negative symptoms of schizophrenia and an improved response in a variety of psychiatric symptom scales in patients, compared to treatment with haloperidol (22).

Further SGAs have been developed since olanzapine was discovered including quetiapine (AstraZeneca) and risperidone (Janssen Pharmaceuticals Inc.), which differ slightly in their receptor binding affinities (23). Aripiprazole is an SGA that was developed by Otsuka Pharmaceutical Co., Ltd. and approved for use in the early 2000s (24). It is unique from the other SGAs as it is a partial agonist of D2 and 5-HT1A receptors and an antagonist of 5-HT2A receptors (14). Aripiprazole has been labelled a ‘dopamine stabiliser’ as its partial agonistic properties allow for treatment of hypodopaminergia and hyperdopaminergia of different brain regions that contribute to negative and positive symptoms (25). Despite aripiprazole’s alternative mode of action, it has been found to have a similar efficacy to D2 receptor antagonists and a lower efficacy when compared to olanzapine, although it is better tolerated by patients (26).

### The risk of developing metabolic syndrome is 3-fold in patients taking antipsychotic medication

While SGAs do not have adverse motor symptoms, they are not without other side effects which reduce a patient’s tolerability of the drug. SGAs can have a sedative effect on patients, they can cause endocrine issues such as sexual dysfunction as well as prominent metabolic side effects (27). The incidence of metabolic syndrome (MetS) is significantly higher in patients taking SGAs (32–68%) than in SGA-naïve patients (3.3–26%) (28). MetS describes a co-occurrence of morbidities such as hypertension, obesity, dyslipidaemia and peripheral insulin resistance which are predictors of type 2 diabetes and cardiovascular disease (CVD) (29, 30). Psychosis alone is a known risk factor for developing MetS (31). Treatment with SGAs exacerbate metabolic symptoms which, along with lifestyle changes and genetic predisposition, increase the risk of CVD (31, 32). The main cause of mortality in schizophrenia patients is CVD, suggesting treatment with SGAs contribute to a patients decline in cardiovascular health and increased risk of CVD-related mortality (33).

Olanzapine and clozapine are the most efficacious antipsychotics, proven to be the only antipsychotics to reduce the risk of suicidality in patients (34, 35), but they are also associated with the most severe risk of MetS. From a meta-analysis, clozapine was shown to be associated with the highest amount of weight gain in patients, and olanzapine with the greatest degree of BMI increase in patients (36). Aripiprazole is associated with the lowest risk of weight gain and metabolic abnormalities (36, 37). The mechanisms behind the increased propensity for SGAs to trigger MetS are largely unknown. Due to their diverse receptor binding profiles, there is the potential for SGAs to bind to receptors in the periphery or alternative off-target binding sites which leads to metabolic disturbances. This off-target binding may include receptors or proteins in the liver, pancreas, kidneys or adipose tissue.

### Mitochondrial stress and antipsychotic-associated metabolic effects

**Figure 1:**
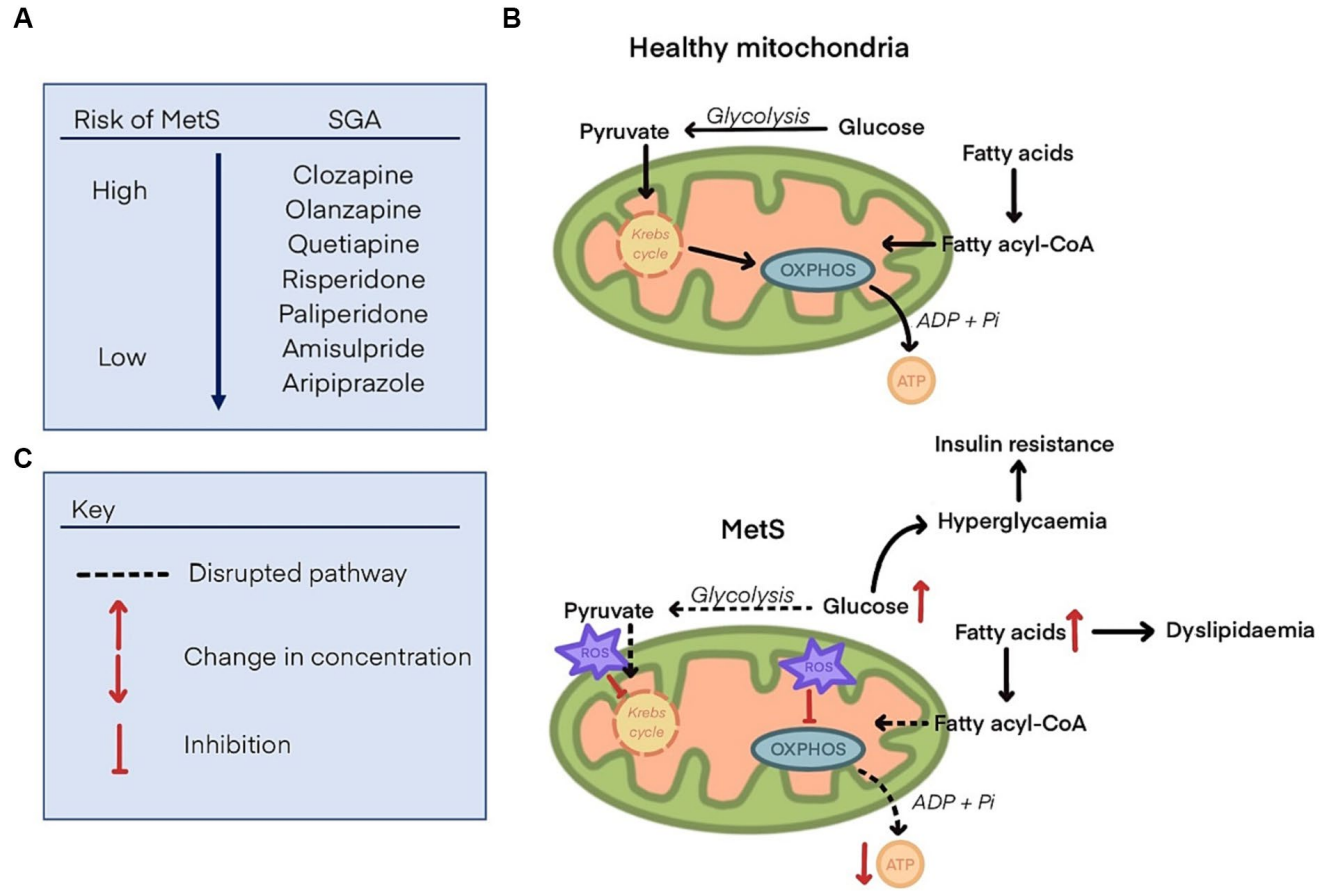
**(A)** List of second-generation antipsychotics and their propensity to cause metabolic syndrome, ranging from the highest risk (clozapine), to the lowest (aripiprazole). **(B)** Glucose and fatty acid metabolism to generate substrates that fuel oxidative phosphorylation (OXPHOS) and produce ATP in health mitochondria. **(C)** Disrupted glucose and fatty acid metabolism in mitochondria with metabolic syndrome. Reactive oxygen species (ROS) are increased and inhibit components of the Krebs cycle and OXPHOS, leading to a decline in ATP synthesis. Glucose and fatty acids concentrations are increased, leading to dyslipidaemia, hyperglycaemia, and insulin resistance.

Mitochondrial dysfunction is a hallmark of metabolic dysregulation and the development of MetS (38). Fundamentally, the disparity between energy production and utilisation can lead to metabolic disorders (39). Mitochondria are responsible for energy production via ATP synthesis, the main energy source for cells. Disruption of this energy production, potentially by inhibition of mitochondrial proteins responsible for ATP synthesis, is likely to either lead to metabolic disorders or may be a consequence of such. Oxidative stress in mitochondria may be at least partly responsible for the pathogenesis of insulin resistance, weight gain and CVDs (40). Other disruptions to regular mitochondrial functioning include scarcity of substrates required to fuel glycolysis or oxidative phosphorylation (OXPHOS) which are processes by which ATP is generated. This may be due to a reduced capacity for uptake of nutrients into cells or inhibition of enzymes involved in the synthesis of substrates (41) (summarised in Figure 1). Therefore, one theory is that SGAs impair mitochondrial function which leads to the development of metabolic disorders by disrupted energy metabolism (42).

### Antipsychotics induce dysregulation of glucose and fatty acid metabolism

Glucose metabolism is shown to be impaired in patients taking SGAs independent of adiposity and age. In one study, the highest blood glucose and insulin resistant levels were found in patients taking olanzapine and clozapine, while risperidone and FGA-treated patients did not show increased insulin resistance but did have elevated glucose levels (43). As patients were matched by adiposity and age, this decline in cardiovascular health and changes in glucose metabolism can be primarily attributed to treatment with olanzapine and clozapine. Further evidence for this was shown when mice were administered olanzapine and they showed an increase in body weight and adiposity that was not concurrent with the rate of food consumption (44).

Antipsychotics have also been shown to disrupt fatty acid metabolism (45, 46). Fatty acid oxidation yields more ATP than glucose but consumes more oxygen (47). A series of studies in mice and rats found that olanzapine lowered plasma free fatty acid (FFA) concentrations and lowered respiratory exchange ratio (RER) prior to fasting, suggesting that olanzapine treatment triggers a shift towards fatty acid oxidation to produce fuel substrates for respiration (45, 46). This switch to fatty acid oxidation as a fuel source rather than glucose, leads to accumulation of glucose and glucose intolerance. C57BL/6 mice were treated a single dose of olanzapine, clozapine, risperidone and aripiprazole (doses between 1 and 10 mg/kg) and the general rate of oxygen consumption and RER was measured using indirect calorimetry (45). Olanzapine, clozapine and risperidone reduced the rate of oxygen consumption and RER dose-dependently, and in olanzapine-treated mice this was associated with an increase in plasma glucose concentration, while aripiprazole showed a much more reduced effect on RER lowering. Similar results were found in Sprague–Dawley rats who were given 10 mg/kg of olanzapine over 24 h (46).

The effects of SGAs on AMP-activated protein kinase (AMPK) signalling has been investigated as an explanation for increases in plasma glucose levels and weight gain (48–52). AMPK senses energy in response to the AMP/ATP ratio and an increase in AMP suggests that ATP is being used up for energy processes in cells at a faster rate than it is being synthesised. AMPK regulates cellular metabolism and in its active form, phosphorylated AMPK, it stimulates glucose uptake and fatty acid oxidation in the liver but AMPK signalling in the hypothalamus reduces glucose uptake and lipid metabolism (53, 54). The differential downstream effects of AMPK, according to the effected tissue, may provide an insight into the off-target metabolic effects of SGAs. SGAs have been shown to have a varying effect on AMPK expression (summarised in Table 1), depending on the tissue studied. Overall olanzapine, clozapine and quetiapine increase AMPK signalling in brain regions such as the hypothalamus and frontal cortex (49–52). As for the effect of SGAs on AMPK signalling in peripheral tissues such as the liver and adipocytes, the results are conflicting (44, 48, 55, 56).

**Table 1:** Summary of the effects of SGAs on AMPK expression.

Author and year	Model cell/organism	Tissue/organ	Antipsychotic medication(s)	Effect on AMPK	Downstream effects
Stapel et al. (55)	PBMCs	-	Olanzapine and aripiprazole	Olanzapine down-regulated AMPK, aripiprazole had no effect	Reduced glucose uptake into cells
Ikegami et al. (50)	ICR mice	Hypothalamus	Olanzapine	Increased AMPK expression in olanzapine-treated mice	Glucose intolerance in mice, increase in plasma glucose levels and insulin levels
Schmidt et al. (44)	C57BL/6 J mice	Liver	Olanzapine	Activated AMPK	Enhanced glycolysis, increased hepatic lipid accumulation
Oh et al. (48).	C57BL/6 mice Primary hepatocytes from Sprague–Dawley rats	Liver	Olanzapine and clozapine	Olanzapine decreased AMPK signalling in primary hepatocytes. Clozapine treatment in mice slightly reduced AMPK phosphorylation	Reduced oxidation of fatty acids and promoted hepatic lipid accumulation
Li et al. (56)	3 T3-L1 adipocytes	-	Olanzapine	Decreased phosphorylated AMPK	Upregulation of SREBP pathway and adipogenesis
Kim et al. (51)	Sprague–Dawley rats	Frontal cortex	Clozapine	Activated AMPK	Downstream inhibition of acetyl CoA carboxylase. Activation of CPT1c which transfers long-chain fatty acyl CoA to mitochondria for beta oxidation
He et al. (49)	Sprague–Dawley rats	Hypothalamus	Olanzapine	Increased AMPK expression via blockade of hypothalamic H1 receptor	Hyperphagia in rats
Okada et al. (52)	Primary astrocytes from neonatal Sprague–Dawley rats Sprague–Dawley rats	Hypothalamus	Quetiapine	Increased phosphorylated AMPK at therapeutic doses	-

### Antipsychotics increase reactive oxygen species and oxidise proteins involved in metabolism

Clozapine and olanzapine have been shown to increase production of reactive oxygen species (ROS) and oxidise key proteins involved in energy metabolism (57–61). ROS are produced by complex I and III during oxidative phosphorylation when leaked electrons react with oxygen to form superoxide ions (62). ROS damage protein structure and function by oxidation. Complex I of the electron transport chain has been shown to be particularly sensitive to ROS attack (63). An increase in the production of ROS leads to damage of mitochondrial elements, a positive feedback of ROS production and consequently, impaired metabolism. As antipsychotics interact with dopamine receptors, their interference with normal dopamine metabolism has been shown to favour the formation of hydrogen peroxidase through monoamine oxidase (58). Using 6-iodoacetamid fluorescein (IAF) labelling which is not incorporated into cysteine residues following protein oxidation, clozapine-treated neuroblastoma cell line SKNSH cells showed increased protein oxidation (57). The proteins were identified using HPLC-electrospray ionisation tandem mass spectrometry and were shown to be a variety of mitochondrial enzymes including malate dehydrogenase, pyruvate kinase and 3-oxoacid CoA. Malate dehydrogenase and pyruvate kinase were also found to be oxidised by olanzapine using cDNA microarray technology on olanzapine-treated rat frontal cortices (59). These enzymes catalyse the oxidation of malate and production of pyruvate which are substrates in the Krebs cycle that facilitate the reduction of NAD+ to NADH to feed electrons into complex I.

Clozapine treatment induces morphological changes in mitochondria in neuroblastoma cells, insulin-responsive and obesity-associated cell types when cultured with TMRM and visualised with confocal microscopy (60). The morphological changes, including mitochondrial swelling and depolarisation, observed with clozapine treatment were similar to those reported in obese rodent models and patients with MetS. It was suggested that the mitochondrial swelling may be in response to an increase in ROS production as a protective mechanism and that the swelling led to depolarisation which was consistent with a depletion of ATP production.

Mitochondrial membrane potential (MMP) loss suggests components of the ETC are dysfunctional, potentially due to the accumulation and damage by ROS (64). In isolated rat hepatocytes, olanzapine induced overproduction of ROS as indicated by DCFH-DA dye, which hydrolyses to DCFH in the liver and fluorescent DCF when interacting with ROS. Fluorescent intensity was then measured using a spectrofluorometer revealing significantly increased fluorescence in olanzapine-treated hepatocytes (61). The cationic fluorescent dye rhodamine 123 was used to measure mitochondrial membrane potential (MMP). This dye accumulates in intact mitochondria and an increase in fluorescence indicates the loss of MMP. MMP loss was found in olanzapine-treated hepatocytes using this method. MMP is a result of the chemiosmotic proton gradient supported by complexes of the ETC and is important for ATP synthesis through ATP synthase.

### The effect of antipsychotics on electron transport chain complexes: conflicting evidence

The effects of antipsychotics on complexes of the ETC has shown differing results. One study found a range of antipsychotics inhibited respiration through specific complexes (65). By measuring oxygen consumption of isolated pig brain mitochondria exposed to antipsychotics, they identified a range of antipsychotics that inhibited complex I-linked respiration. Haloperidol, risperidone, quetiapine and aripiprazole fully inhibited complex I-linked respiration over a range of doses and clozapine was reported to almost fully inhibit complex-I linked respiration. A smaller range of doses also inhibited the activity of complex II, III and IV. However, amongst these antipsychotics, olanzapine, one of the most potent APs associated with metabolic side effects, did not significantly inhibit mitochondrial respiration in this study. There is numerous evidence to support that Haloperidol and other FGAs inhibit complex I of the ETC and this has been implicated in the development of EPS (66). However, whether metabolic abnormalities triggered by SGA treatment is as a result of ETC complex inhibition has not been fully explored.

Lymphoblastoid cell lines (LCLs) isolated from schizophrenia patients taking clozapine, olanzapine, aripiprazole, quetiapine and healthy controls have also yielded metabolic information (42). LCLs treated with clozapine, olanzapine and quetiapine significantly reduced complex I and II activity and decreased mitochondrial respiration, with olanzapine having the most severe effect (42). There was reduced (though not statistically significant) activity of complex I and II in untreated cells from schizophrenia patients compared to controls. This study produced very different results and suggests that metabolic dysfunction in schizophrenia patients may be associated with olanzapine reducing efficiency of mitochondrial respiration. It also suggests that schizophrenia patients may be predisposed to developing MetS and subsequent treatment with SGAs triggers further metabolic decline through inhibition of mitochondrial complexes. Indeed, a common genetic risk factor for schizophrenia, a deletion at chromosome 3q29, has been associated with mitochondrial dysfunction (67). There was a reduced ability to transition from glycolysis to OXPHOS during neuronal maturation in human cortical organoids, downregulation of complex II and IV of the ETC in mice brains with the 3q29 deletion and an overall increased ‘vulnerability to metabolic challenges’ in these models.

## Conclusion

Here, we have reviewed whether the prominent metabolic side effects seen with SGA treatment, primarily with olanzapine and clozapine, are related to mitochondrial dysfunction that results in impaired metabolism. Considering the efficacy of clozapine and olanzapine in treating psychosis relative to drug alternatives and non-pharmacological interventions, determining the cause of the metabolic impairments is of significant interest. Establishing the best method for treatment through optimal polypharmacy or personalised medicine, will have a considerably beneficial outcome on psychosis patients’ quality of life in the short term. In the long term, the development of new antipsychotics with a reduced risk of metabolic side effects should be the aim.

## Author contributions

KM: Conceptualization, Writing – review & editing, Writing – original draft. MK: Conceptualization, Supervision, Writing – review & editing. LC: Conceptualization, Writing – review & editing, Funding acquisition, Supervision.
